# The number of Estonian black fungus gnats (Diptera, Sciaridae) doubled: the first records of 64 species

**DOI:** 10.3897/BDJ.12.e123368

**Published:** 2024-06-14

**Authors:** Ina Gorban, Kai Heller, Olavi Kurina

**Affiliations:** 1 Vilnius university, Vilnius, Lithuania Vilnius university Vilnius Lithuania; 2 Senckenberg, Müncheberg, Germany Senckenberg Müncheberg Germany; 3 Unaffiliated, Heikendorf, Germany Unaffiliated Heikendorf Germany; 4 Institute of Agricultural and Environmental Sciences, Tartu, Estonia Institute of Agricultural and Environmental Sciences Tartu Estonia

**Keywords:** Sciaridae, Estonian black fungus gnats, biodiversity

## Abstract

**Background:**

Adult sciarid flies are small to medium-sized, delicate insects, typically black in colour and belonging to the superfamily Sciaroidea within the order Diptera. They exhibit a uniform appearance. Distinguishing them from other families of Sciaroidea is primarily achieved through their typical wing venation. Sciaridae are common in both natural and semi-natural ecosystems, where they play a significant role in decomposition during their larval stage. Prior to the current study, only one specific research project had been conducted on Sciaridae in Estonia. The number of sciarid species identified in Estonia was set at 67.

**New information:**

This research, conducted in north-eastern Estonia during 2015 and 2016, presents a comprehensive overview of Sciaridae diversity, shedding light on previously understudied aspects of Estonia's biodiversity. A total of 1038 specimens were identified, representing 91 species, amongst which 64 were previously unknown to Estonia. Notably, *Corynopterawinnertzi* (Mohrig, 1993) emerged as the most abundant species, followed by *Corynopterairmgardis* (Lengersdorf, 1930), *Corynopteracrassistylata* (Frey, 1948) and *Bradysiatrivittata* (Staeger, 1840). The genus *Corynoptera* Winnertz, 1867 exhibited the highest diversity, consistent with findings from broader European studies. This study underscores the importance of ongoing surveys for better understanding the distribution and composition of Sciaridae species in Estonia, emphasising the need for further research to delve into the intricate ecology and biology of these insects.

## Introduction

The Sciaridae, commonly known as black fungus gnats, is one of the most diverse groups of flies, with a global distribution and a significant presence in Europe. These flies are typically small to medium-sized, ranging from 0.8 to 7.0 mm in body length and have long legs and antennae. While the family can be distinguished by the characteristic venation of their wings, identifying individual species can be challenging due to their similar appearance (Menzel and Mohrig 2000).

Adult Sciaridae are commonly found in shaded environments such as forests and swamps, while their larvae typically inhabit soil, leaf litter or dead wood (Irmler et al. 1996, Hövemeyer and Schauermann 2003, Seeber et al. 2012, Gorban and Podėnienė 2022). The larvae of Sciaridae are important in soil ecology, as they feed on fungi, decaying plant matter and bacteria. The black fungus gnats are known for their ecological importance as decomposers, contributing to the breakdown of organic matter in soil and other substrates (Menzel and Schulz 2007, Broadley et al. 2018, Menzel et al. 2020). Despite their ecological and economic significance, the taxonomic and molecular diversity of Sciaridae remains poorly understood.

In Europe, 700 species of sciarids have been registered (Vilkamaa 2014), but the fauna of sciarids in Estonia remains poorly studied. Menzel et al. (2020) summarised our knowledge of Estonian Sciaridae by listing six species covered by earlier authors and, along with two species described as new to science, added 55 new country records. However, they overlooked a local publication by Remm (1959) covering eight species from the Avaste bog in western Estonia. Of these eight species, four were included subsequently also by Menzel et al. (2020), viz. *Bradysiatrivittata* (Staeger, 1840), Cratyna (Spathobdella) nobilis (Winnertz, 1867) – Remm (1959), as *Bradysia*, *Ctenosciarahyalipennis* (Meigen, 1804) – Remm (1959), as *Leptosciaraautumnalis* Winn. and Phytosciara (Dolichosciara) ornata (Winnertz, 1867) – Remm (1959), as *Leptosciara*. An additional four species were not recorded by Menzel et al. (2020), viz. *Bradysiavernalis* (Zetterstedt, 1851), *Corynopteratristicula* (Winnertz, 1867) – Remm (1959), as *Bradysia*, Scatopsciara (Scatopsciara) humeralis (Zetterstedt, 1851) – Remm (1959), as *Lycoria* and *Sciarahemerobioides* (Scopoli, 1763) – Remm (1959), as *Lycoriathomae* L. Thus, the number of Estonian Sciaridae species has been set at 67 prior to the current study.

## Materials and methods

**Figure 1.**
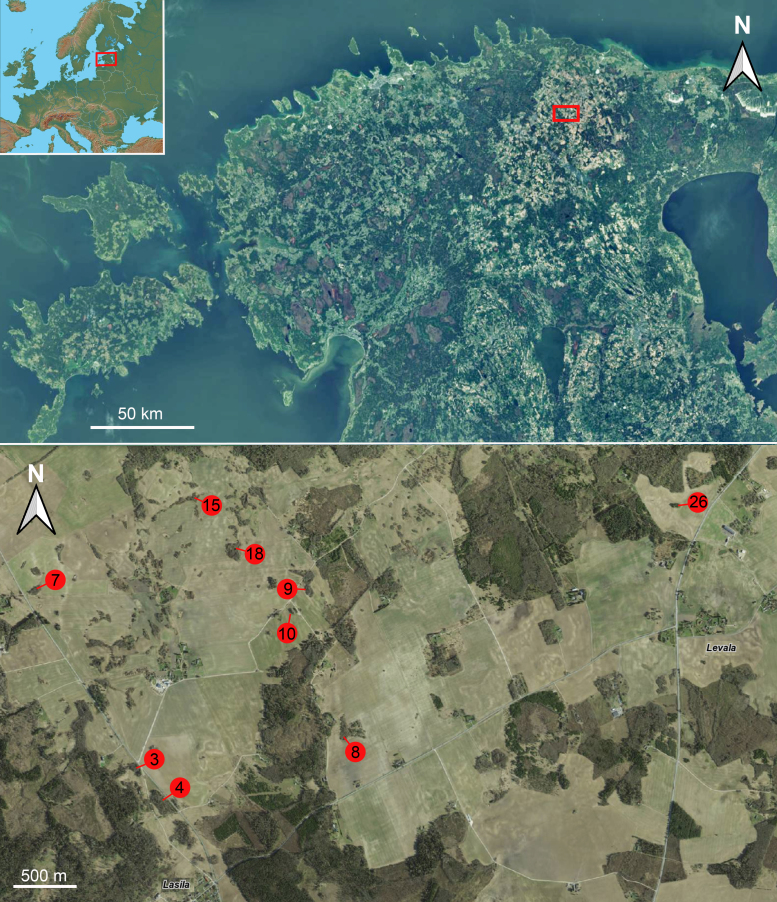
Collecting localities are defined as forest patches within agricultural landscape. The numbers of forest patches correspond to those in Takkis et al. (2018). Maps of Estonia: Estonian Land Board (https://xgis.maaamet.ee/xgis2/page/app/maainfo; accessed 31.03.2023).

**Figure 2.**
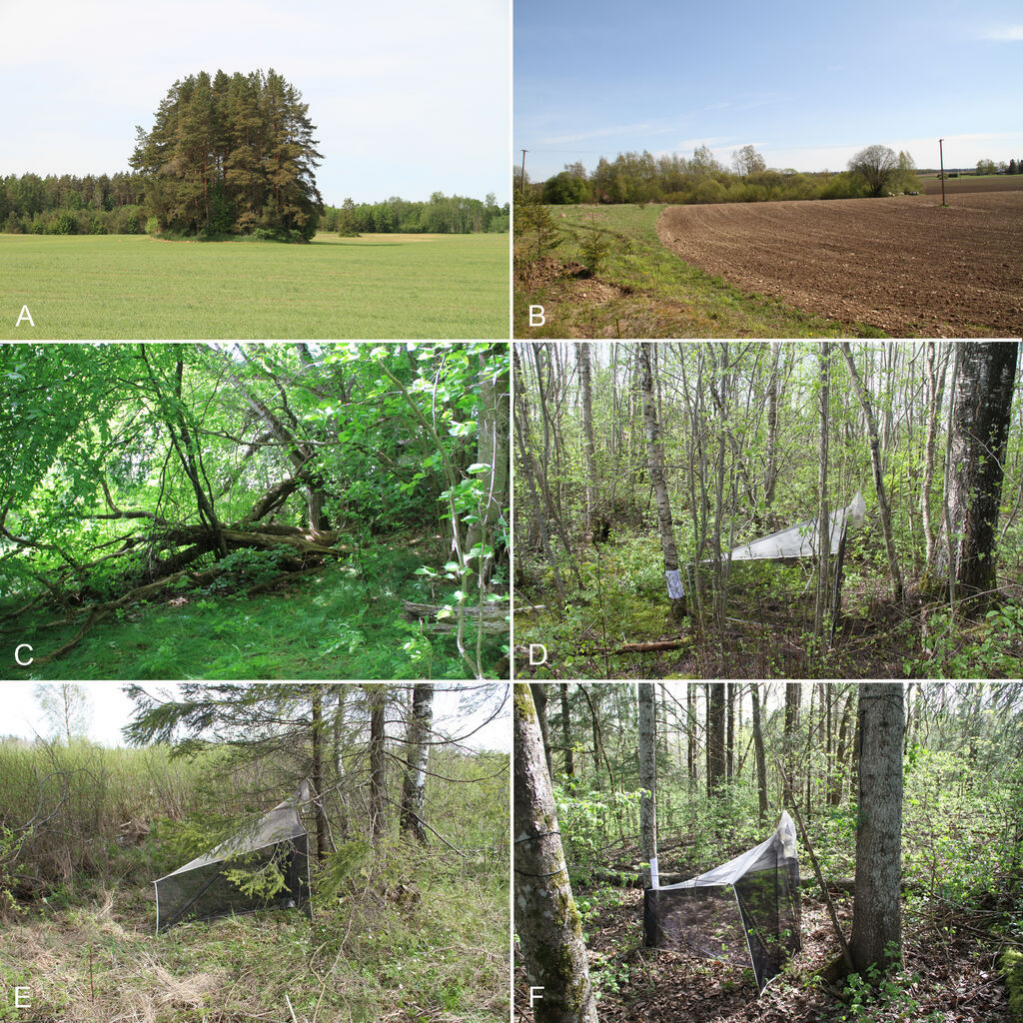
A selection of sampled forest patches and installed Malaise traps (MT). **A** Forest patch No. 26 is dominated by conifers and surrounded by arable land; **B** Forest patch No. 8 is temporarily wet and dominated by brush-wood; **C** Forest patch No. 18 harbours a lot of decaying wood; **D** MT set up in forest patch No. 8; **E** MT set up in forest patch No. 15; **F** MT set up in forest patch No. 4. Photos by O. Kurina.

All material of the present communication was collected with Malaise traps. We used "Czech type" traps from Ento Sphinx (http://www.entosphinx.cz/) with reconstructed collecting heads (see Tomasson et al. (2014)). The traps were operated in north-eastern Estonia in 2015 and 2016 during biodiversity monitoring of forest patches embedded amongst agricultural landscape. Nine such patches (Fig. 1), which varied in size, vegetation type and structure (Fig. 2), were sampled. The species richness and its determinants of vascular plants were studied by Takkis et al. (2018) and the numbers of forest patches (Fig. 1) correspond to that analysis. The material was collected in about 70% ethyl alcohol and then sorted into selected groups including Sciaridae.

Moulded slides were prepared for the identification of most Sciaridae male specimens. However, some were identified under a binocular microscope due to their visible differences, making moulded slides unnecessary. For the slide preparation, a small drop of Euparal, a mounting medium, was placed on to a clean microscope slide. The intact specimen was then carefully transferred on to the drop of Euparal and a coverslip was placed over it. These prepared slides were examined under a microscope, allowing for detailed observation of key morphological features.

The studied material was catalogued and deposited in the Kai Heller private collection, in the collection of Life Sciences Center of Vilnius University, Lithuania and in the insect collection IZBE – Institute of Agricultural and Environmental Sciences, Estonian University of Life Sciences (former Institute of Zoology and Botany), Tartu, Estonia.

The Simpson’s index of diversity for forest patches was calculated using the software EstimateS, Version 9.1.0. (Colwell 2013).

## Data resources

The voucher specimens data underpinning our research are available from a public dataset at https://doi.org/10.15156/BIO/2959324 (Kurina 2024). Alternatively, complete voucher specimen information can be accessed as part of the large Institutional dataset in GBIF at https://www.gbif.org/dataset/1af83152-24f7-4df7-afbc-b213b62175bb. For the full list of identified material, please refer to the supplementary table (Suppl. material 1).

## Checklists

### Sciaridae collected in North-East Estonia 2015–2016

#### Sciaridae

Billberg, 1820

A391EE23-0B2B-58C1-8799-9159A82269A3

##### Notes

A total of 1038 specimens were identified, representing 91 species, amongst which 64 were previously unknown in Estonia (Table 1). The genus *Corynoptera* Winnertz, 1867 was found to be the most diverse with 25 species, followed by *Bradysia* Winnertz, 1867 with 15 species and *Leptosciarella* Tuomikoski, 1960 with nine species. The remaining genera showed relatively low diversity, with only 1 - 6 species each.

## Discussion

Our research presents a comprehensive and updated overview of the diversity and distribution of Sciaridae species in Estonia, which was previously understudied. The results highlight a greater diversity of fungus gnats in Estonia than previously thought, underscoring the importance of ongoing surveys to enhance our understanding of the composition and distribution of these insects.

Our research highlights the dominance of the genus *Corynoptera*, which was found to be the most diverse with 25 species in Estonia. The high abundance of *Corynoptera* species is consistent with previous studies, which have shown that this genus is commonly found in Europe (Salmela and Vilkamaa 2005, Hippa et al. 2010, Vilkamaa et al. 2013, Babytskiy et al. 2019).

Significantly, our study revealed that the majority of identified species were represented by 10 or fewer individuals, whereas only a few species had more than 50 individuals. Amongst the recorded species, 29 and 19 were represented by singletons and doubletons, respectively. This suggests a much higher species richness and emphasises the necessity of surveying a large number of individuals to fully capture the diversity of insect populations, as rare species may be easily overlooked. When comparing the different forest patches, most of them have Simpson's index of diversity (D) over 0.8, indicating a high probability that two randomly collected individuals belong to different species (Table 2). Surprisingly, collecting in the smallest forest patch (No. 10; 0.07ha) resulted in the highest number of singletons and unique species for the project (D = 0.94). This phenomenon is intriguing, given that the habitat is quite dry, nearly devoid of decaying wood and open to the wind. Conversely, forest patch No. 18, which contains significant amounts of decaying wood and wet microhabitats, also possesses the same Simpson's index of diversity (D = 0.94).

## Supplementary Material

XML Treatment for Sciaridae

F0F64996-9F7F-5B36-94F6-DE573EB064A1 10.3897/BDJ.12.e123368.suppl1 Supplementary material 1 Estonian Sciaridae Data type Occurrence data Brief description The list of all studied specimens of Sciaridae collected from north-eastern Estonia, 2015-2016. File: oo_1052327.xls https://binary.pensoft.net/file/1052327 Gorban I, Heller K, Kurina O

## Figures and Tables

**Table 1.** Sciaridae collected in north-east Estonia 2015–2016. The numbers of forest patches correspond to those in Fig. 1 and in Takkis et al. (2018). Abbreviations: * = First record from Estonia; *Cra.* = *Cratyna* s. str.; *Pey.* = *Peyerimhoffia*; *Spa.* = *Spathobdella*.

Species	Forest patch number									Total no. of specimens
	3	4	7	8	9	10	15	18	26	
*Bradysiaaffinis* (Zetterstedt, 1838)*	1			1						2
*Bradysiaarcula* Vilkamaa, Salmela & Hippa, 2007*	1	1					1			3
*Bradysiabreviallata* Mohrig & Menzel, 1992*		1		1						2
*Bradysiaexcelsa* Menzel & Mohrig, 1998*	2					1				3
*Bradysiafenestralis* (Zetterstedt, 1838)*						1			1	2
*Bradysiafungicola* (Winnertz, 1867)*	1									1
*Bradysiahercyniae* (Winnertz, 1869)*	1									1
*Bradysiahilariformis* Tuomikoski, 1960*	2					1				3
*Bradysiainusitata* Tuomikoski, 1960*			1							1
*Bradysialobulifera* Frey, 1948*						1				1
*Bradysiapectoralis* (Staeger, 1840)				1						1
*Bradysiapilistriata* Frey, 1948*			1							1
*Bradysiatilicola* (Loew, 1850)			1							1
*Bradysiatrivittata* (Staeger, 1840)	5		15	2	25	6	2	3	3	61
*Bradysiavagans* (Winnertz, 1868)*			8	4	6		2			20
*Camptochaetacamptochaeta* (Tuomikoski, 1960)								2	1	3
*Claustropygabrevichaeta* (Mohrig & Antonova, 1978)								2		2
*Claustropygasubcorticis* (Mohrig & Krivosheina, 1985)*								1		1
*Corynopterablanda* (Winnertz, 1867)*					3				1	4
*Corynopteraboletiphaga* (Lengersdorf, 1940)				1	26	14		4	1	46
*Corynopteracrassistylata* (Frey, 1948)*	45				46					91
*Corynopteracurvata* Mohrig & Mamaev, 1987*					2					2
*Corynopteradefecta* (Frey, 1948)*					2					2
*Corynopterafatigans* (Johannsen, 1912)*				1						1
*Corynopteraflavicauda* (Zetterstedt, 1855)*	4	1								5
*Corynopteraforcipata* (Winnertz, 1867)	1			2			1			4
*Corynopterafurcifera* Mohrig & Mamaev, 1987*			1	2	2					5
*Corynopterainvoluta* (Frey, 1948)*	3	1	1	3	1		6	4		19
*Corynopterairmgardis* (Lengersdorf, 1930)	7		34	52	2	8	7	5		115
*Corynopteramelanochaeta* Mohrig & Menzel, 1992							1	1		2
*Corynopteraobscuripila* Tuomikoski, 1960*	2				8			1	1	12
*Corynopteraparvula* (Winnertz, 1867)*	3		6			1	4	5		19
*Corynopteraparvulaformis* Mohrig, 1985*						2				2
*Corynopterapiniphila* Lengersdorf, 1940*									13	13
*Corynopterapraeforcipata* Mohrig & Mamaev, 1987*						3				3
*Corynopterasaetistyla* Mohrig & Krivosheina, 1985						2				2
*Corynopterasubdentata* Mohrig, 1985*					1					1
*Corynopterasubforcipata* Mohrig & Menzel, 1990*				2						2
*Corynopterasubparvula* Tuomikoski, 1960*						1				1
*Corynopterasubtilis* (Lengersdorf, 1929)*	6	1		1	3	7			3	21
*Corynopteratridentata* Hondru, 1968					3	2		3		8
*Corynopteraunidentata* (Hippa & Vilkamaa, 1994)	8			8	9	4	1	13	3	46
*Corynopterawinnertzi* Mohrig, 1993*	310	1						1	1	313
Cratyna (Cra.) ambigua (Lengersdorf, 1934)*					1					1
Cratyna (Cra.) cryptospina (Rudzinski, 1993)*			1							1
Cratyna (Cra.) pernitida (Edwards, 1915)*	1							1		2
Cratyna (Pey.) vagabunda (Winnertz, 1867)*			4	1	2	6	1	10	1	25
Cratyna (Spa.) falcifera (Lengersdorf, 1933)			7	3	1	1	1	2	1	16
Cratyna (Spa.) nobilis (Winnertz, 1867)			4	1	1	2		1		9
*Ctenosciarahyalipennis* (Meigen, 1804)						1				1
*Dichopyginaintermedia* (Mohrig & Krivosheina, 1982)*						1				1
*Dolichosciaraflavipes* (Meigen, 1804)*				1						1
*Epidapusatomarius* (De Geer, 1778)*						1		1		2
*Epidapusgracilis* (Walker, 1848)*					1			1		2
*Epidapusmicrothorax* (Börner, 1903)*								1		1
*Epidapusschillei* (Börner, 1903)*							1			1
*Leptosciariellabrevipalpa* (Mohrig & Menzel, 1992)						2				2
*Leptosciarielladimera* (Tuomikoski, 1960)*			1							1
*Leptosciariellafuscipalpa* (Mohrig & Mamaev, 1979)						5			1	6
*Leptosciariellarejecta* (Winnertz, 1867)*	1	1	1							3
*Leptosciariellascutellata* (Staeger, 1840)*						1		1		2
*Leptosciariellasubpilosa* (Edwards, 1925)					1					1
*Leptosciariellasubspinulosa* (Edwards, 1925)*				1						1
*Leptosciariellatrochanterata* (Zetterstedt, 1851)*					1					1
*Leptosciariellayerburyi* (Freeman, 1983)*			3	1			4	1		9
*Leptospinatruncata* (Tuomikoski, 1960)*			1			1		1		3
*Lycoriellaacutostylia* Mohrig & Menzel, 1990*			1	10			1			12
*Lycoriellabrevipila* Tuomikoski, 1960*					1	5	1	1		8
*Lycoriellaconspicua* (Winnertz, 1867)*			1							1
*Lycoriellalundstromi* (Frey, 1948)	2									2
*Lycoriellamicria* Mohrig & Menzel, 1990*	1									1
*Prosciaraprosciaroides* (Tuomikoski, 1960)			1	2						3
*Pseudolycoriellabrunnea* (Bukowski & Lengersdorf, 1936)*					3			10		13
*Pseudolycoriellasubbruckii* (Mohrig & Hövemeyer, 1992)			1							1
*Scatopsciaraatomaria* (Zetterstedt, 1851)	1		3	1			1			6
*Scatopsciaracalamophila* Frey, 1948				1			1	2	1	5
*Scatopsciaraedwardsi* Freeman, 1983*	1							1		2
*Scatopsciarafritzi* Mohrig & Menzel, 1992*						1				1
*Scatopsciaravitripennis* (Meigen, 1818)*			11							11
*Sciaraflavimana* Zetterstedt, 1851*						1				1
*Sciarahebes* (Loew, 1870)			1		2					3
*Sciarahemerobioides* (Scopoli, 1763)					1					1
*Sciararuficauda* Meigen, 1818*					1					1
*Trichosiacaudata* (Walker, 1848)							2	1		3
*Trichosiaconfusa* Menzel & Mohrig, 1997	1				1					2
*Trichosiaedwardsi* (Lengersdorf, 1930)*		1			1	1				3
*Trichosialengersdorfi* Heller, Köhler & Menzel, 2016*				1					1	2
*Trichosiasplendens* Winnertz, 1867*							1	1	2	4
*Xylosciaraheptacantha* Tuomikoski, 1957*								2	2	4
*Xylosciaramisella* (Frey, 1948)*	1				2					3
*Zygoneurasciarina* Meigen, 1830*	1			3		1				5

**Table 2.** Basic data of the sampled forest patches near Lasila, north-east Estonia along with summarised results of the collected Sciaridae. The Simpson’s index of diversity is calculated using the following equation: D = 1 - ∑ n (n-1) / N (N-1) where *n* represents the number of individuals of each species in all samples of a specific trap and *N* is the total number of specimens of all species collected by that trap. The index is not calculated for forest patch No. 4 because the trap was repeatedly destroyed during the collecting period, rendering the data incomparable. The forest patch area and distance are sourced from Takkis et al. (2018).

Forest patch number	**3**	**4**	**7**	**8**	**9**	**10**	**15**	**18**	**26**
Coordinates	59.2613N, 26.2088E	59.2591N, 26.2115E	59.2742N, 26.1955E	59.2634N, 26.2379E	59.2735N, 26.2338E	59.2721N, 26.2312E	59.2804N, 26.2181E	59.2768N, 26.2229E	59.2786N, 26.2853E
Area (ha)	0.56	1.75	0.41	0.82	1.06	0.07	0.73	1.23	0.13
Distance to closest patch (m)	14	30	30	10	10	16	18	8	151
Collected specimens	412	8	109	107	158	84	39	83	37
Species	26	8	24	26	30	30	19	30	17
Singletons per Malaise trap	13	8	14	14	13	16	12	16	11
Singletons per project	3	0	7	4	6	6	1	2	0
Unique species per project	4	0	7	5	8	10	1	3	1
Simpson’s index of diversity	0.42	NA	0.86	0.75	0.86	0.94	0.93	0.94	0.87
